# FORMIDABEL: The Belgian Ants Database

**DOI:** 10.3897/zookeys.306.4898

**Published:** 2013-06-03

**Authors:** Dimitri Brosens, François Vankerkhoven, David Ignace, Philippe Wegnez, Nicolas Noé, André Heughebaert, Jeannine Bortels, Wouter Dekoninck

**Affiliations:** 1 Instituut voor Natuur en Bosonderzoek, Kliniekstraat 25, B-1070, Brussels, Belgium; 2 Polyergus, Wolvenstraat 9, B-3290, Diest, Belgium; 3 FourmisWalBru, Rue Winston Churchill 91, B-6180, Courcelles, Belgium; 4 FourmisWalBru, Rue de la Grotte, 23, B-4651, Herve, Belgium; 5 ULB, Université Libre de Bruxelles, Campus de la Plaine CP257, Brussels, Belgium; 6 ULG, Université de Liège, Gembloux Agro-Bio Tech, Passage des Déportés, 2, B-5030 Gembloux - Belgium; 7 Royal Belgian Institute of Natural Sciences (RBINS), Vautierstraat 29, B-1000, Brussels, Belgium

**Keywords:** Formicidae, Belgium, Flanders, Wallonia, Brussels Capital Region, ecological data, grid mapping, UTM, historical data, literature, collections, observations, trapping, ants

## Abstract

FORMIDABEL is a database of Belgian Ants containing more than 27.000 occurrence records. These records originate from collections, field sampling and literature. The database gives information on 76 native and 9 introduced ant species found in Belgium. The collection records originated mainly from the ants collection in Royal Belgian Institute of Natural Sciences (RBINS), the ‘Gaspar’ Ants collection in Gembloux and the zoological collection of the University of Liège (ULG). The oldest occurrences date back from May 1866, the most recent refer to August 2012. FORMIDABEL is a work in progress and the database is updated twice a year.

The latest version of the dataset is publicly and freely accessible through this url: http://ipt.biodiversity.be/resource.do?r=formidabel. The dataset is also retrievable via the GBIF data portal through this link: http://data.gbif.org/datasets/resource/14697

A dedicated geo-portal, developed by the Belgian Biodiversity Platform is accessible at: http://www.formicidae-atlas.be

**Purpose:** FORMIDABEL is a joint cooperation of the Flemish ants working group “Polyergus” (http://formicidae.be) and the Wallonian ants working group “FourmisWalBru” (http://fourmiswalbru.be). The original database was created in 2002 in the context of the preliminary red data book of Flemish Ants (Dekoninck et al. 2003). Later, in 2005, data from the Southern part of Belgium; Wallonia and Brussels were added. In 2012 this dataset was again updated for the creation of the first Belgian Ants Atlas (Figure 1) (Dekoninck et al. 2012). The main purpose of this atlas was to generate maps for all outdoor-living ant species in Belgium using an overlay of the standard Belgian ecoregions. By using this overlay for most species, we can discern a clear and often restricted distribution pattern in Belgium, mainly based on vegetation and soil types.

## Taxonomic coverage

### General taxonomic coverage description

The taxonomic coverage (Figure 2) of this database spans the full range of ants pertaining to Belgium (indigenous ant species and exotic introduced species). The determination level is species level and, if appropriate, hybrid level. For some species, information on micro-and macrogynes is available. Key milestones of FORMIDABEL from conception till date are described in the ”Dataset” section of this manuscript.

**Figure 1.**
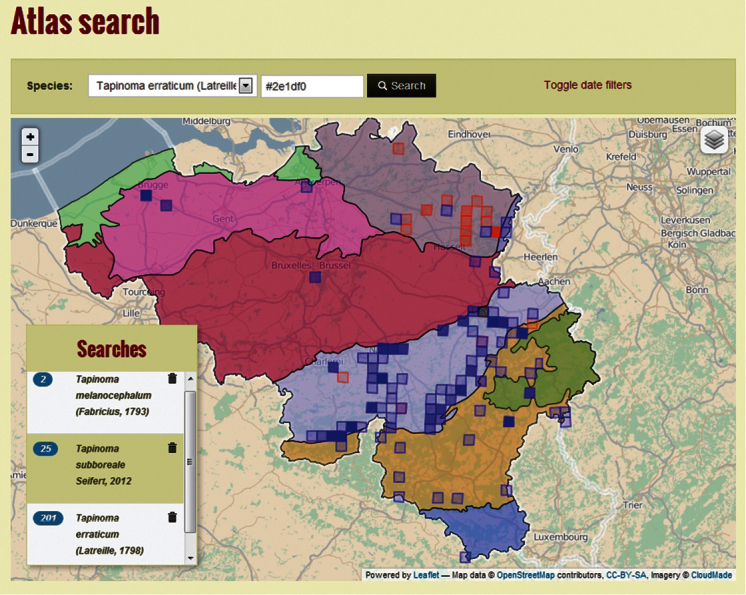
www.formicidae-atlas.be

**Figure 2.**
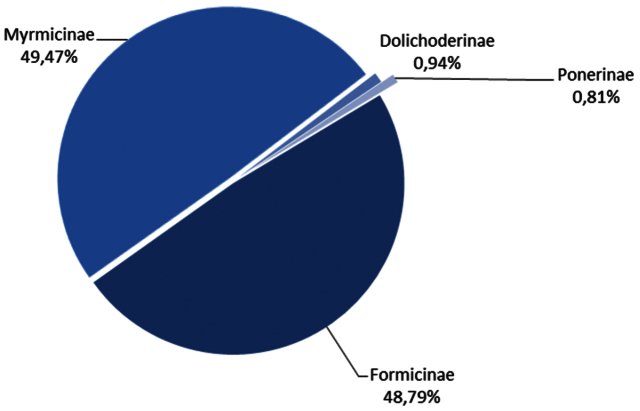
Taxonomic range of the FORMIDABEL database subfamilies

The taxonomic authorities followed are: Radchenko and Elmes (2010) for the genus *Myrmica* and Seifert (2007) for the other genera. The dataset contains occurrences of 76 native and 9 introduced species.

As depicted in Figure 2, the most abundant subfamily in the database is the Formicinae (49,4%) followed by the Myrmicinae (48,7%), the Dolichoderinae (0,9%) and the Ponerinae (0,8%). The top five most recorded species are *Lasius niger* (2846 records), *Myrmica rubra* (2601 records), *Myrmica scabrinodis* (1626 records), *Formica fusca* (1467 records) and *Myrmica sabuleti* (1202 records).

## Taxonomic ranks

**Phylum**: Arthropoda

**Subphylum**: Hexapoda

**Class**: Insecta

**Order**: Hymenoptera

**Suborder**: Apocrita

**Superfamily**: Vespoidea

**Family**: Formicidae

**Subfamily**: Dolichoderinae, Formicinae, Myrmicinae, Ponerinae

**Genera**: *Anergates*, *Aphaenogaster*, *Camponotus*, *Dolichoderus*, *Formica*, *Formicoxenus*, *Harpagoxenus*, *Hypoponera*, *Lasius*, *Leptothorax*, *Linepithema*, *Manica*, *Monomorium*, *Myrmecina*, *Myrmica*, *Plagiolepis*, *Polyergus*, *Ponera*, *Solenopsis*, *Stenamma*, *Strongylognathus*, *Tapinoma*, *Technomyrmex*, *Temnothorax* and *Tetramorium*.

**Common names:** Ants

## Spatial coverage

### General spatial coverage

Belgium is a small country in Western Europe. To the west, its 70 km coastline fronts the North Sea; to the north lies the Netherlands; to the east, Germany, and to the south, France and Luxembourg. Biologeographically, the fauna of eastern Belgium belongs to the Central European Province of the Eurasian (Palaearctic) region. By contrast, the rest of the country primarily consists of an Atlantic fauna plus a few Central European relict species.

**Figure 3.**
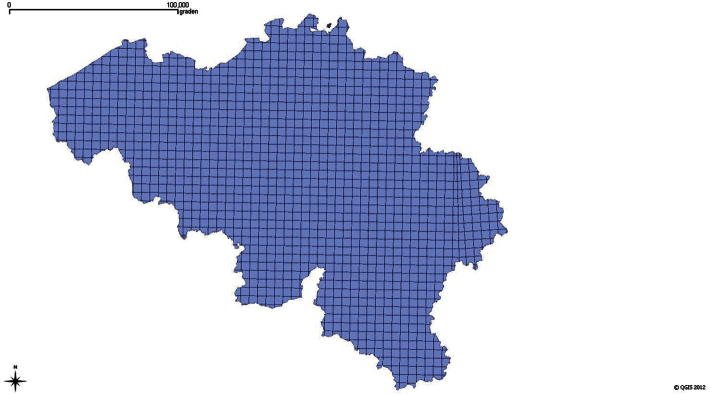
The UTM 5 Km grid of Belgium

Politically and geographically, the country is divided into three parts: Flanders, Wallonia and the Brussels Capital Region (Figure 3). In Flanders (13,522 km² and population about 6 million people), to the north, soils are mainly sandy to loamy. Here, the most important habitats for ants are heathlands and dry grasslands. The Brussels Capital Region is a small region (162 km²) entirely situated in the sandy loam area. In Wallonia (17,006 km² and about 3,5 million people), to the south, soils and habitats are more diverse, ranging from forests to rocky and calcareous grasslands on loam and chalky soils. Eastern Wallonia, near the German border, includes the Hautes Fagnes, a large area of bogs and peat with some typical ant species.

### Geographical method

The Universal Transverse Mercator Projection (UTM), an adaptation of the standard Mercator projection, uses a two dimensional Cartesian co-ordinate system to identify locations on the surface of the Earth (Wikipedia).

**Figure 4.**
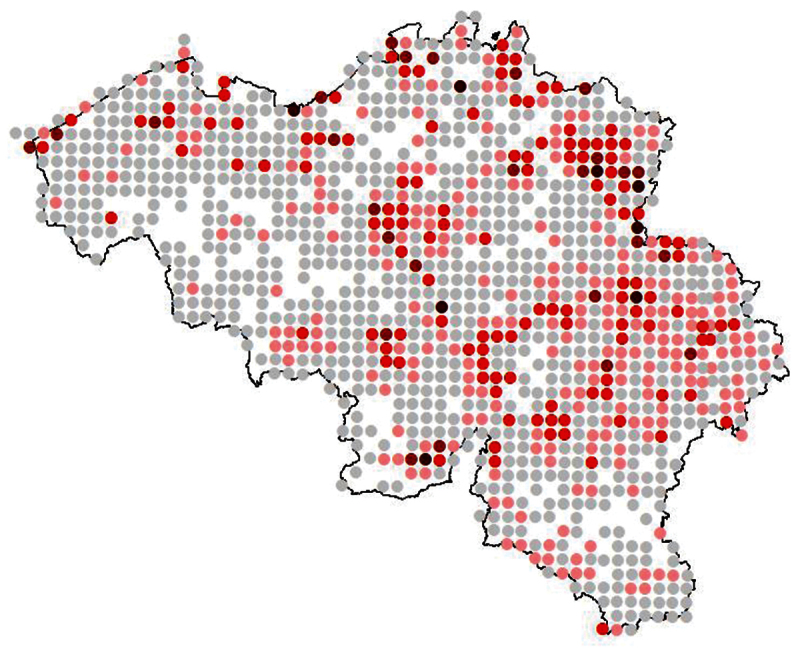
Projection of the number of records per UTM 5x5 km square (grey dots= 1-20 records, pink dots= 21-50 records, red dots= 51-150 records, dark red dots=151-300 records and black dots=301-644 records).

The UTM 5 Km (Universal Transverse Mercator Projection) raster projection divides Belgium in approximately 1200 25 km² squares (Figure 3). A representative number of UTM squares has been sampled (1125 UTM 5×5 km squares of which 659 squares with more than 10 records: see Figures 3 and 4) to complete the dataset. All the records in FORMIDABEL are georeferenced through the centroid coordinates of the corresponding UTM 5 km square. Therefore, the uncertainty on these coordinates is 3.500 meters, the distance between the centre and the corner of the UTM square.

### Ecocodes

More than half of the records are provided with a description of the microhabitat of the record locality. This allows us to give details on ecological preferences of all Belgian ant species. In FORMIDABEL we created a list of potential microhabitats for ants in Belgium. For each of these microhabitats we use a code called the “ecocode”. This code thus gives information on the habitat were the occurrence was made. Nine types of habitat and landscape were defined to collect accurate information on the habitat preference of all ant species (Dekoninck et al. 2005). These nine types are based on the EIS-code and the Flemish nature types (Vandenbussche 2002; Zwaenepoel et al. 2002). When no habitat description was available for a record (for example, with some older records) the habitat was coded as ‘Not known or not observed’. The nine main habitats we defined were: i) anthropogenic habitats, ii) dry grasslands, iii) moist grasslands, iv) forests, v) chalk grasslands, stony slopes and other rocky xerothermic habitats, vi) shrubs, vii) heathlands, viii) fens and highland bogs and ix) coastal and inland dunes.

### Coordinates

49°27'0"N and 51°32'24"N Latitude; 2°28'12"E and 6°27'36"E Longitude

## Temporal coverage

**Figure 5.**
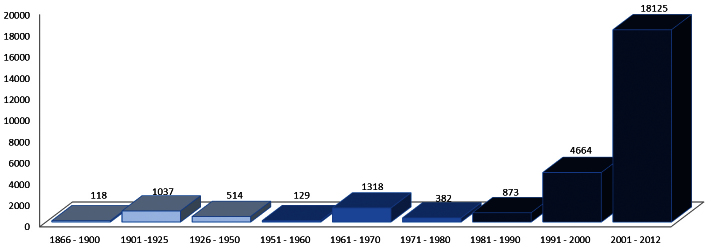
Temporal distribution of the records

The oldest record in the database goes back to May 5, 1866 and the most recent records are from August 2012. The largest part of the records were obtained after 1991 (Figure 5).

## Methods

### Method step description:

A large portion of the occurrence data have been collected by volunteers, other records originated from several projects and research programs. The data and specimens were sent to the Belgian ant curators, and after validation, the information was incorporated in the database. The collection records “dry specimen” originate from the Gembloux “Ant” collection and the Charles Gaspar collection, the collection of the “Cercle des entomologists Liégeois”, the RBINS collection and the private collection “Roland Vannieuwenhuyse”. After revision and validation, this information was also included in the database. The literature-based records were retrieved from van Boven 1970; van Boven and Mabelis 1986; Dekoninck et al. 2006 and references therein. How the database evolved is described in the Database history section.

**Sampling description:** Most occurrence records originate from hand/nest sampling (42,3% of all records and mainly from Wallonia) andpitfall sampling (36,7% mainly from Flanders). The followed procedure differs from region to region. This is due to historical reasons. Some very interesting occurrence records were obtained by sifting, coloured water traps and Malaise traps (all less than 3 % of the total sampling). An extensive description of the sampling methods is provided by Schauff (2001).

**Quality control description:** All the records were validated by the dataset curators before being added to the FORMIDABEL database. The dataset curators also checked the determinations of the collection specimens. If needed, the determination was adapted and made consistent with modern taxonomy; Radchenko and Elmes (2010) for the genus *Myrmica* and Seifert (2007) for the other genera. Before the final publication of the database all the records were tested on geographical consistency by the Belgian Biodiversity Platform and corrected if necessary.

## Dataset

### Dataset history

At the beginning of 2001 all available records of ants in Flanders (northern part of Belgium) were brought together for the first time and several inventories were started. More than 20.000 records (for the most part gathered after 1990) were assembled in the FORMIDABEL (FORMIcidaeDAtaBELgium) database resulting in the ‘Verspreidingsatlas en voorlopige Rode lijst van de mieren van Vlaanderen; Dekoninck et al. 2003. [Distribution atlas and preliminary Red list of ant species in Flanders, Belgium]. This report contains all available distribution data for Flanders. In the southern part of Belgium (Wallonia) intensive inventorying started in 2005. Until then knowledge on the distribution of ants in Wallonia was limited. Only a few areas (Haute Fagnes, Famenne and the Viroin valley) had already been inventoried. Thanks to the good cooperation between the Polyergus and the FourmisWalBru working groups; many Belgian ant records were brought together in the FORMIDABEL database. Since then, FORMIDABEL is updated with data originating from FourmisWalBru twice a year. The FORMIDABEL dataset was then used for the creation of the “Belgian Ant Atlas”, (Dekoninck et al. 2012). In 2010 a cooperation agreement between the authors of the atlas and the Belgian Biodiversity Platform (www.biodiversity.be) was made. Together with the publication of the book, the data was published in Darwin Core Archives to GBIF (url: http://data.gbif.org/datasets/resource/14697/) and a dedicated data portal was created: www.formicidae-atlas.be.

**Figure 6.**
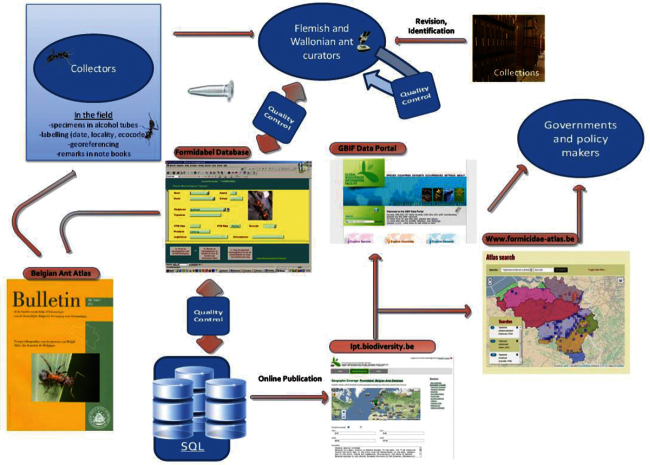
Formidabel flowchart

The original FORMIDABEL database was created in Microsoft Access. Later, this database was completely imported in a relational SQL database. During this process additional data cleaning was performed; see section Quality control description. The dataflow is illustrated in Figure 6.

### Dataset description

The FORMIDABEL Darwin Core Archive is a custom made SQL view on the original version of the FORMIDABEL access database which is in the custody of the Belgian Ant working groups Polyergus and fourmisWalBru. Mind that every record in FORMIDABEL represent at least one occurrence, but primarily contains information on the presence of a species. The view only shows data that are accepted for publication. Fields given are:

id, decimalLatitude, family, basisOfRecord, stateProvince, identifiedBy, eventDate, modified, country, individualCount, scientificName, kingdom, order, geodeticDatum, genus, collectionCode, decimalLongitude, samplingProtocol, catalogNumber, phylum, recordNumber, countryCode, coordinatePrecision, language, coordinateUncertaintyInMeters, locality, specificEpithet, recordedBy, institutionCode, nomenclaturalCode, class.

**Figure 7.**
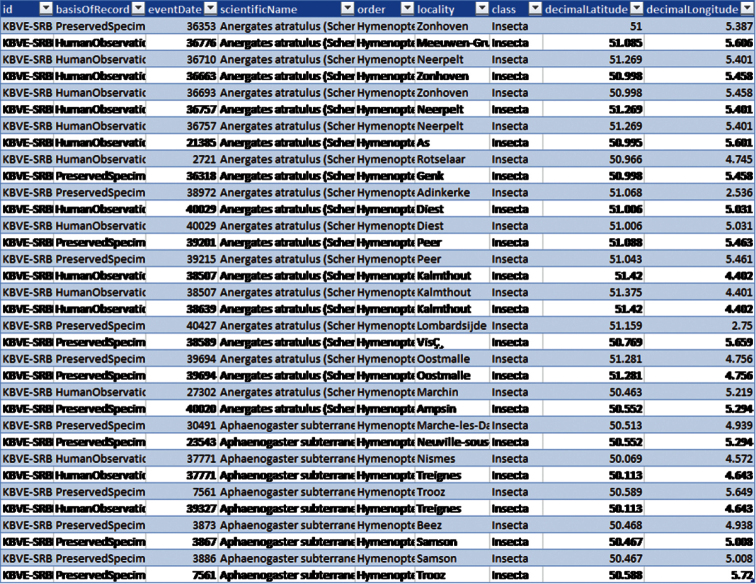
A small preview of the Darwin Core-Archive

The dataset contains primary biodiversity data, mostly occurrence data (Figure 7). Some records hold an indirect link to collection specimens. This link is only available in the original database.

## Dataset preview

**Object name:** Darwin Core Archive Formidabel; Belgian Ants Database

**Character encoding: UTF-8Format name:** Darwin Core Archive format

**Format version:** 1.0

**Distribution:** http://ipt.biodiversity.be/resource.do?r=formidabel

**Publication date of data: 2013-08-02**

**Language: English**

**Licenses of use:** This work is licensed under a Creative Commons Attribution-NonCommercial-ShareAlike 3.0 Unported License. http://creativecommons.org/licenses/by-nc-sa/3.0/

**Metadata language:** English

**Date of metadata creation:** 2013-02-18

**Hierarchy level:** Dataset

**Norms for data use and publication:**

Based on http://www.canadensys.net/norms

Give credit where credit is due

As is common practice in scientific research, cite the data you are using.

Be responsible

Use the data responsibly. The data are published to allow anyone to better study and understand the world around us, so please do not use the data in any way that is unlawful, harmful or misleading. Understand that the data are subject to change, errors and sampling bias. Protect the reputation of the data publisher and clearly indicate any changes you may have made to the data.

Share knowledge

Let us know if you have used the data. It helps our participants to showcase their efforts and it helps you reach a wider audience. Inform the data publisher(s) if you have comments about the data, notice errors, or want more information.

Respect the data license

Understand and respect the data license or waiver under which the data are published. It is indicated in the rights field of every record and in the dataset metadata.

**Collection data:** Ant Collection Gembloux (urn:lsid:biocol.org:col:33368), Collection Charles Gaspar, Collection “Cercle des entomologists Liégeois”, RBINS Belgian Formicidae Collection (urn:lsid:biocol.org:col:35271), Private collection “Van Nieuwenhuyse”. All collections are dry prepared insect collections. The dataset does not contain unique identifiers for specimens. To track a collection specimen, the corresponding author should be contacted.

## Additional information

This dataset was originally created to develop the Belgian Ants Atlas. However, the dataset can be reused for a variety of purposes. Since the link between individual data records and underlined specimens (stored in multiple collections) is not recorded, we doubt if the dataset can be used for taxonomic or systematic studies. However, this being an occurrence dataset, it can be used for understanding species richness, distribution pattern and modeling studies such as ecological niche modeling. In order to enhance the confidence of use, we have documented the metadata as well as subjected the data records to a series of quality assessment and enhancement processes as described in the earlier section quality control description.
